# Promoting Respectful Maternity Care by Reducing Unnecessary Episiotomies: Experiences from Centers of Excellence for Breastfeeding in Vietnam

**DOI:** 10.3390/healthcare11182520

**Published:** 2023-09-12

**Authors:** Duong Hoang Vu, Binh T. T. Ta, Ina Landau Aasen, Dai Q. T. Le, Roger Mathisen, Genevieve E. Becker, Hang Thi Phan, Cuong Minh Bui, Trinh Thi Kieu Nguyen, Suong Thi Thu Hoang, Jennifer Cashin

**Affiliations:** 1 Alive & Thrive East Asia Pacific, FHI Solutions, Hanoi 11022, Vietnamrmathisen@fhi360.org (R.M.); 2 Norwegian Institute of Public Health (NIPH), N-0473 Oslo, Norway; 3 BEST Services, H91 T22T Galway, Ireland; 4 Hung Vuong Hospital, Ho Chi Minh City 72700, Vietnam; 5 Quang Ninh Obstetrics and Pediatrics Hospital, Ha Long City 01100, Vietnam; 6 Quang Nam Provincial General Hospital, Tam Ky 51100, Vietnam; 7 Phu Vang District Hospital, Hue 49900, Vietnam; 8 Alive & Thrive East Asia Pacific, FHI Solutions, Washington, DC 20037, USA

**Keywords:** episiotomy, vaginal childbirth, early essential newborn care, guideline, quality of care, breastfeeding, Vietnam, policy

## Abstract

(1) Background: Routine episiotomy is not recommended by international guidelines; however, it occurs at a high rate in Vietnam. (2) Methods: A process to reduce unnecessary episiotomies was developed and implemented as part of the Centers of Excellence for Breastfeeding initiative, which aims to deliver high-quality breastfeeding and early essential newborn care services within a supportive policy environment. The aim of this project report is to outline the steps undertaken to reduce episiotomies, the experience in pilot hospitals, and the process towards changing policy. (3) Results: During the 14 months following the change in episiotomy policy, pilot hospital records showed no infant death or injury. Monthly monitoring data from four pilot hospitals showed that the prevalence of episiotomy was substantially lower than the average in national hospitals in Vietnam. Facilitators to reducing the episiotomy rate include the incentive of Centers of Excellence for Breastfeeding designation and supportive hospital leadership. Challenges include the ambiguity of Vietnam’s national guideline on episiotomy and lack of routine monitoring on the episiotomy rate and indications. (4) Discussion: Our experience suggests that through training and routine monitoring hospitals can apply a policy of selective episiotomy and reduce the practice, particularly among multiparous women, and improve breastfeeding rates.(5) Conclusions: Sharing our experience of implementing this process and offering four areas for action will hopefully contribute to expanded use of mother-friendly, evidence-based care as policy and routine practice in Vietnam and similar settings.

## 1. Introduction

Episiotomy is an incision made in the perineum during childbirth to widen the vaginal opening. While the procedure was common in the past, current guidance on evidence-based care indicates that episiotomy should not be performed routinely, is not justified by clinical trial and evidence review literature [1,2], and is not recommended by either the World Health Organization (WHO) [3,4,5] or obstetric and midwife professional associations [6,7]. As reviewed in the WHO guidance [3,4,5], episiotomy can negatively impact maternal emotional well-being, cause perineal trauma and pain, infection, longer healing time, difficulty with baby care and breastfeeding, delayed infant bonding, delayed breastfeeding, decreased sexual enjoyment, and fear of future births. As negative experiences of episiotomies are shared among women, these accounts may contribute to a climate of doubt and fear about giving birth. In addition to the human impact, unnecessary episiotomies increase health service costs due to the need for equipment, suturing materials, analgesic and management of possible infections.

Despite this evidence, episiotomy continues to be routine practice in many countries in the Western Pacific region. As shown in Table 1, data from postpartum mothers’ interviews conducted in 161 randomly selected health facilities implementing early essential newborn care show large variation in episiotomy rates by country: from 0% in Papua New Guinea and the Solomon Islands to 71% in Vietnam [8]. A recent cross-sectional household survey of mothers of infants in three diverse settings in Vietnam showed a similarly high prevalence of episiotomy. Among women giving birth vaginally, 83% reported receiving an episiotomy, and those who received the procedure were significantly less likely to initiate breastfeeding within the first hour as recommended [9].

Countries in the region use different means of data collection. New Zealand reported episiotomy rates of 17% for all vaginal births (2019) [10], and Australia reported 23% for non-instrumental births and 80% for instrumental births [11] involving the use of forceps, a vacuum, or another instrument to extract the fetus from the vagina, with or without concurrent maternal pushing. Norway reported episiotomy for 36.5% of primiparous women and 8.3% of multiparous women who gave birth vaginally during the period of 2015–2017, including instrumental births. For non-instrumental vaginal births, the reported episiotomy rate was 22.7% for primiparous women and 5.7% for multiparous women during the same period [12]. 

These variable rates of episiotomy suggest that many are performed for reasons other than clinical need. High rates of routine episiotomy despite lack of evidence on benefits may be related to misperceptions and faulty beliefs among health care providers and pregnant women. A study in a large Cambodian hospital with an episiotomy rate over 90% reported that the “reasons given for this practice by midwives and obstetricians were: fear of perineal tears, the strong belief that Asian women have a shorter and harder perineum than others, lack of time in overcrowded birthing facilities, and the belief that Cambodian women would be able to have a tighter and prettier vagina through this practice” [13]. Similarly, a survey among obstetricians and midwives in one Vietnam hospital with a high rate found that the primary reason episiotomies were implemented was an attempt to minimize 3rd and 4th degree perineal tears [14]. However, research has found that episiotomy does not reduce the occurrence of severe perineal tears [15], and that Asian women do not have a differently structured perineum compared to non-Asian women [16]. 

Recognition of the high and varied rates of episiotomy resulted in a priority action to address unnecessary use of episiotomy across nine countries during the WHO Western Pacific third biennial meeting on implementation of early essential newborn care (EENC) [17]. This priority action increased awareness about the importance of this issue and added to the determination of health authorities at the national level to reduce episiotomy rates.

For women who are expected to have a spontaneous vaginal birth, a policy of selective episiotomy may result in 30% fewer experiencing severe perineal/vaginal trauma. A study in Shanghai, China found that mothers with selective episiotomy had no increased risk of severe perineal/vaginal trauma, and a lower risk of postpartum hemorrhage [18]. Recent clinical trials in Israel and Brazil have found no difference in maternal or perinatal outcomes between mothers randomized into no-episiotomy, selective-episiotomy, or standard care groups, highlighting the need to explore whether there is any indication for the procedure [2,19]. 

According to the WHO, “Shifting from a policy of routine or liberal to selective/restrictive use of episiotomy will require a change in organization culture, training, monitoring and continuous clinical practice audit” [4]. Respectful maternity care policies show promise for improving women’s experience of care and reducing incidents of abusive care. A recent systematic review found evidence (albeit at a low level of certainty) of an association between adoption of respectful maternity care policies in hospital environments and reduced episiotomy rates [20]. Ensuring that women have a companion of choice during birth has also been shown to reduce unnecessary caesarean sections and episiotomies [21]. 

Nearly 1.5 million babies are born each year in Vietnam, 96% of whom are born in health facilities [22]. Adoption of policies to reduce unnecessary medical procedures and improve patient satisfaction can have a substantial impact on service access and quality at a population level. This project report describes preliminary results from an intervention by the Ministry of Health and select hospitals in Vietnam collaborating with Alive & Thrive (A&T). The objective of the intervention was to test whether training, hands-on coaching, and the introduction of routine monitoring is effective at facilitating changes in obstetric and midwifery procedures to reduce the routine practice of episiotomy, as one aspect of a broader initiative to facilitate a positive birth experience. This report includes a description of the intervention, routine monitoring data from participating hospitals, and reflection on the role of policy and guidance, capacity development of health professionals, communication among pregnant women and the general community, and evidence generation in facilitating changes in the practice of episiotomy.

## 2. Materials and Methods

### 2.1. Program Description

**Figure 1:**
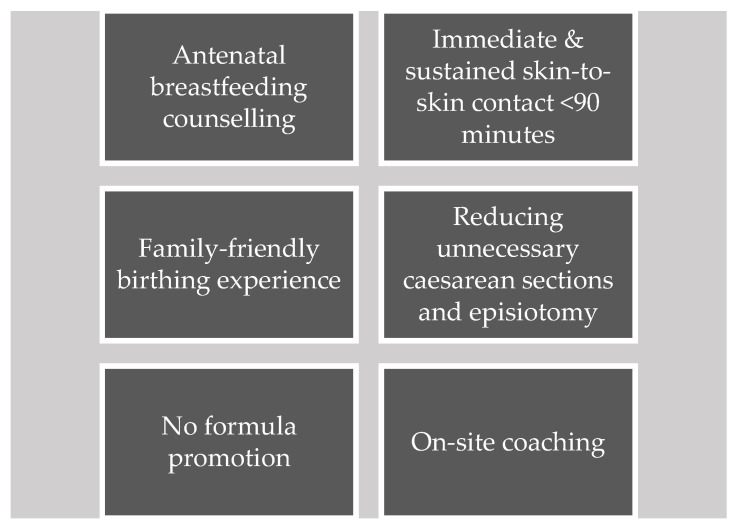
Approaches to improve provider-driven behaviors in support of breastfeeding and respectful maternity care.

A&T is an initiative to save lives, prevent illness, and ensure healthy growth and development of mothers and infants [23]. In the East Asia Pacific region, A&T provides strategic technical assistance to governments and a network of local partners with a focus on eight countries (including Vietnam). The aim of this technical assistance is to facilitate policy and system reforms to increase investment and create an enabling environment for nutrition. The Centers of Excellence for Breastfeeding (COE) [24,25] initiative, designed and launched by A&T in 2019, strengthens the capacity of health systems in Cambodia, Laos, and Vietnam to deliver high-quality breastfeeding and EENC services within a supportive policy environment. Services include breastfeeding counselling and support as well as the consistent implementation of practices that support breastfeeding, namely: immediate and sustained skin-to-skin contact, non-separation of the mother–baby dyad, no promotion of commercial milk formula, and reduction in unnecessary medical interventions that disrupt breastfeeding, including routine episiotomy and caesarean section (Figure 1). The COE initiative is informed by a strategy to (1) incentivize health facilities to improve performance by identifying end-enrolling champion hospitals in the initiative and creating healthy competition and (2) generate demand for breastfeeding-friendly, respectful maternity care services among families through strategic communications on social and traditional media [25]. 

### 2.2. Episiotomy Guidelines Development

Practical episiotomy guidelines were prepared reflecting guidance from the WHO Western Pacific Regional Office (WPRO) clinical practice guide [26] and the findings of a Cochrane systematic review in 2017 [1]. A&T compared Vietnam’s National Technical Guideline on episiotomy (2013) [27] with the WHO WPRO clinical practice guide. Based on this, practical selective episiotomy guidelines for hospitals were developed (Table 2). 

### 2.3. Training Session Development

A&T collaborated with the Norwegian National Advisory Unit on Breastfeeding, Oslo University Hospital to prepare the two-day Family-Friendly Childbirth Room and Skilled Birth Attendance training of trainers (TOT) program, which included a review of indications for episiotomy and development of skills for reducing unnecessary episiotomies. The learning program was based on the WHO *Recommendations: Intrapartum Care for a Positive Childbirth Experience* [4] and included a half-day presentation on theory, literature review of evidence for recommended practices, the Norwegian case study, and a question–and-answer session. In addition, the program included a half-day practice on a mannikin plus one-day practice with cases of vaginal births and reflections on experiences at the hospital. 

### 2.4. Recruitment

Hospitals (N = 12) were purposively selected for participation based on their enrollment in the COE initiative; they were either leading maternity hospitals with teaching mandates or private hospitals with leaders committed to quality improvement that were specifically recruited for participation (see list of hospitals in Table A1, Appendix A). These private hospitals have lower annual birth rates, leading to lower caseloads for doctors and midwives, which could allow for more rapid provider behavior change. Adoption of selective episiotomy policies in the private sector may also incentivize public hospitals to change their own protocols since both public and private hospitals in Vietnam aim to maximize revenue by attracting more patients covered by the national health insurance scheme through the provision of more patient-friendly services, including respectful maternity care [28]. Among participating hospitals, the total annual number of births ranged from 5000 to 65,000. 

Midwives and medical doctors (N = 117) who were the heads of shifts or held managerial positions were recruited from each hospital and participated in the TOT on Family-Friendly Childbirth Room and Skilled Birth Attendance in November 2019. The hospital directors, deputy directors, or, in the case of general hospitals, head doctors of obstetrics and gynecology departments also attended the course so that they could support their staff in implementing the new protocols. These trainers were expected to conduct courses and clinical coaching in their own hospitals using a similar format of presentation of theory, practice with mannikins, and practice and reflection with actual births. The length of the learning program depended on the needs of each hospital, e.g., implementing a review or introduction of new practices. As the trainers were shift managers or similar, they could provide ongoing coaching for staff as needed.

### 2.5. Data Collection

Vietnam’s Ministry of Health does not require hospitals to routinely monitor and report on the practice of episiotomy. To address this gap, A&T introduced a self-monitoring form with the following variables: episiotomy (yes/no); natural tear (yes/no); no tear (Supplementary Table S1). Hospital-level data collection and a monthly self-monitoring procedure were introduced and included: (1) Individual recording of the reason for episiotomy, if it occurred, in the mother’s medical record; (2) Daily recording of the number and percentage of births with episiotomies, natural tears, and no tear disaggregated by primiparous and multiparous women; and (3) Use of a format to allow subsequent linking to EENC hospital databases as well as COE assessments at hospitals. Monitoring data from 4 of the 12 participating hospitals from March 2020 to April 2021 was used to calculate the average prevalence of episiotomy following the TOT (see Table A1 in Appendix A).

## 3. Results

### 3.1. Episiotomy Usage

**Figure 2:**
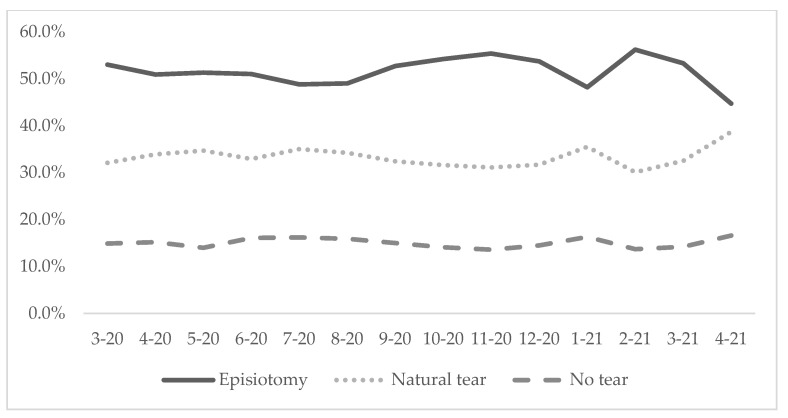
Prevalence of episiotomy, natural tear and no tear in four hospitals from March 2020 to April 2021 (N = 31,541).

In the 14 months following the learning program, hospital records for 31,541 vaginal births in four hospitals showed no infant death or injury, and only one fourth degree natural tear following the change in episiotomy policy. Monthly monitoring data from births in the four hospitals (N = 31,541) showed that the prevalence of episiotomy was substantially lower than the average of 71% in national hospitals of Vietnam [8]. From March 2020 to April 2021, the average episiotomy rate among the four hospitals was reduced from 53.0% to 44.7% (Figure 2).

**Figure 3:**
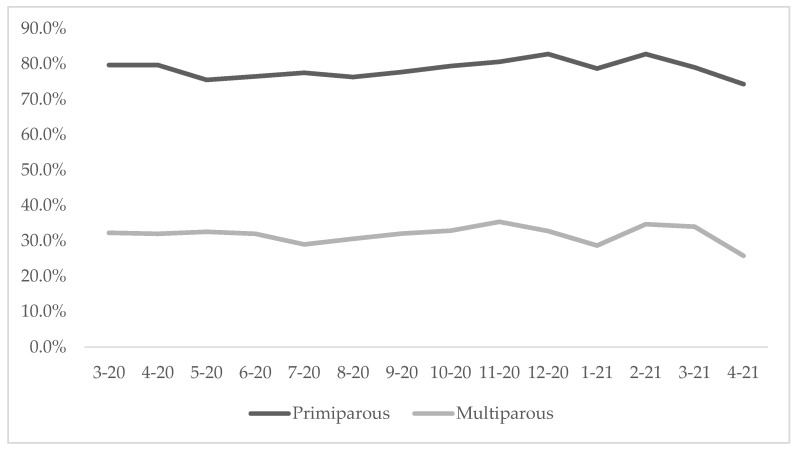
Prevalence of episiotomy among primiparous and multiparous women in four hospitals from March 2020 to April 2021 (N = 31,541).

When we disaggregated data by parity, we found that the episiotomy prevalence among primiparous women was substantially higher (74.3%) than among multiparous women (25.8%) (Figure 3).

### 3.2. Facilitators to Adopting the New Episiotomy Policy into Routine Practice

During semi-structured interviews conducted as a part of an evaluation of the COE initiative, participating health workers stated that practical guidelines on episiotomies and building consensus to apply them were valuable to promote uptake across different departments and shifts. Additionally, the requirement to record the specific episiotomy indication in the mother’s medical record kept a focus on using the new guidelines and tracking the indicators [29]. Participants also indicated that the learning program and support they received from hospital directors and shift managers reduced fears related to the policy change and empowered providers to adopt the new practice. Furthermore, the absence of negative outcomes for infants helped persuade health staff to adopt the new guidance [29]. 

### 3.3. Challenges to Adopting the New Episiotomy Policy

**Figure 4:**
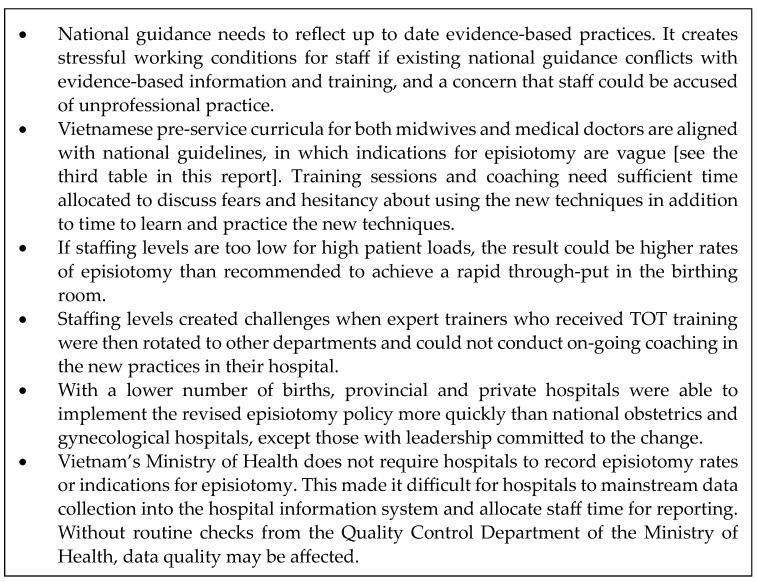
Challenges associated with the adoption of a selective episiotomy policy, based on feedback from training participants.

Twelve hospitals participated in the training of trainers, eight hospitals collected and shared their data, and four of these had complete data. Feedback from participating health workers highlighted challenges implementing the new episiotomy policy [29], some of which could be addressed through training while others require larger solutions, as noted in Figure 4.

### 3.4. Policy Advocacy

Results from A&T’s analysis of Vietnam’s National Technical Guideline on Indications for Episiotomy as stipulated in Decision 1377/QD-BYT [26] are presented in Table 3. Key findings included: (1) indications for performing an episiotomy are not clearly stated in the guidelines; and (2) these indications can be made more specific and adjusted to meet WHO WPRO guidance and align with the practical guidelines in Table 2 above.

In November 2019, A&T sent a letter to the Director General of the Department of Maternal and Child Health (MCH), Ministry of Health (see Supplementary Material S2), presenting the results of this analysis and preliminary results from the learning program described above. The MCH Department responded by initiating an official review of the National Technical Guideline by an expert committee comprised of hospitals that participated in the project and other leading hospitals, a process which is still ongoing.

## 4. Discussion

The project was carried out in 12 birthing health facilities in Vietnam to explore the possibility of reducing unnecessary episiotomies by applying practical selective episiotomy guidelines that were developed based on WHO Western Pacific Regional Office (WPRO) clinical practice standards. Pilot projects like this one provide information on feasibility of a particular intervention and potential challenges. Our findings indicate that changing provider behaviors to promote respectful maternity care within a complex system can be addressed through a reasoned approach that considers the interrelationship between policies and guidance, health care providers, expectant and new mothers and their influencers, local infrastructure, health and information systems, and external influences. This interrelated, reasoned approach is applicable to all birthing facilities in Vietnam and to similar settings in the Western Pacific region, especially health systems with decentralized facility financing that have invested in the scale-up of early essential newborn care [30]. 

Based on the experiences and reflections of this process, we provide recommendation for how an updated episiotomy guideline and practice can be rolled out. Our recommendations are organized by four key areas: policy advocacy, pre- and in-service learning, communication, monitoring and evidence generation:

### 4.1. Policy Advocacy

- Updating the national technical guideline by the Department of Maternal & Child Health to reflect evidence-based practice and clarity in its implementation.
- Allocating ongoing budget for training and coaching for trainers and staff members.
- Working with best practice and teaching hospitals, such as the ones enrolled in the COE initiative and professional organizations of midwives and medical doctors, to recognize the updated guidance as an expected standard of professional practice.

### 4.2. Pre- and In-Service Learning

- Reviewing and including the updated guidance in the pre-service curriculum and clinical experiences for midwives and medical doctors.
- Establishing and sustaining a core group of health workers at each maternity facility to have responsibility for in-service training and ongoing skills coaching and monitoring of practice.

### 4.3. Communication

- Communicating with related and collaborating organizations to use the new episiotomy guideline in their activities.
- Including the recording, monitoring, and audit of episiotomy/tears as routine obstetric data collection and in quality indicators.
- Keeping mothers and fathers and significant influencers aware of the guidance and how it affects the health and well-being of mother and baby, including through media channels.
- Ensuring antenatal information provided to pregnant women includes the guidance and time to discuss how it applies to their individual situation.
- Delivering social and behavior change communication for health workers to increase their confidence and willingness to apply new clinical guidelines.

### 4.4. Monitoring and Evidence Generation

- Conducting qualitative and quantitative research on mothers’ experiences of episiotomy and the impact on their lives after childbirth (e.g., quality of life assessment), using data to develop advocacy messages and materials targeted toward policy makers and service users.
- Carrying out an economic evaluation of the costs of episiotomy, including any negative outcomes, such as infection, arising from the intervention to ascertain the economic aspects of unnecessary episiotomy and savings through reducing rates in order to inform decision-making.
- Updating the health information system to include mandatory reporting on episiotomy rates to mainstream monitoring, improve data quality and compliance with the updated national technical guideline.

## 5. Conclusions

This project report describes our experience of implementing a process to reduce unnecessary episiotomies in select birthing facilities in Vietnam to promote a positive birth experience for mothers and improved mental and physical health for mothers and babies. This process was undertaken as a part of the COE initiative, a recent evaluation of which found that a hospital’s enrollment in the initiative was significantly associated with increased rates of early initiation of breastfeeding and exclusive breastfeeding during women’s stay in the hospital [24]. Our experience suggests that, through training and routine monitoring, hospitals in Vietnam can apply a policy of selective episiotomy and reduce the practice, particularly among multiparous women. Global and country-specific evidence suggests that reducing unnecessary episiotomies will further improve breastfeeding rates [4,9]. However, key barriers to policy and practice change remain. 

There were several limitations to this pilot project. First, project monitoring coincided with the COVID-19 pandemic (March 2020 to April 2021), which led to the de-prioritization of policy changes and reduced the ability of expert trainers to cascade training to health staff in their hospitals due to staff shortages and restrictions on the size of in-person gatherings. Second, routine monitoring of episiotomy is not a requirement for hospitals in Vietnam, so not all participating hospitals continuously monitored their progress after the TOT. Third, our conclusions on the effectiveness of this pilot project are based on self-monitoring data from the hospitals where the authors work. Thus, our results are presented in the form of a project report rather than as original research. 

While acknowledging the above limitations, we are also working on next steps for the program. We will conduct research to further explore the impact of a selective episiotomy policy on maternal quality of life and its cost-effectiveness for health facilities. We believe our experience to date will be valuable to those working to implement and scale up mother-friendly, evidence-based care in Vietnam and other settings.

## Figures and Tables

**Table 1:** Episiotomy rate by country in the Western Pacific region ^1^.

Country	National Hospital (%)	Subnational Level Hospital (%)
Vietnam ^2^	71	70
Lao PDR ^3^	36	30
Cambodia ^2^	3	26
China ^3^	27	25
Mongolia ^3^	11	11
Papua New Guinea ^2^	0	23
Philippines ^2^	29	29
Solomon Islands ^2^	0	27

^1^ Data from 161 randomly selected health facilities implementing EENC in eight countries (National hospitals include 32 national/regional hospitals; Subnational level hospitals include 121 first level referral hospitals and 8 first level hospitals); ^2^ Data from 2017; ^3^ Data from 2016.

**Table 2:** Selective episiotomy guidelines for hospitals.

An episiotomy should only be performed when indicated by:
Abnormal progression of labor * Non-reassuring fetal heart rate pattern Vacuum or forceps childbirth Shoulder dystocia
Episiotomy indications must be written in the medical record
* *Definition of abnormal progression of labor:*
*(a)* *Primiparous women with or without epidural anaesthesia: >3 h* *(b)* *Multiparous women with or without epidural anaesthesia: >2 h*

**Table 3:** A&T analysis of Vietnam’s National Technical Guideline on Indications for Episiotomy as stipulated in Decision 1377/QD-BYT dated 24 April 2013, on National technical guideline for examination and treatment in Gynecology and Obstetrics.

Vietnam’s National Technical Guideline on Indications for Episiotomy	Analysis
1. Maternal causes: - The perineum is thick, hard, swelling due to prolonged labor or many vaginal examinations	“The perineum is thick, hard, swelling” is a vague indication. In many cases, it is due to the limited waiting time for normal labor. A&T proposes to delete the “thick, hard, swelling perineum” indication and keep the indication of “prolonged labor”, which has been defined in the proposed practice guideline indication 1 of “abnormal progression of labor”.
- Maternal disease: heart failure, high blood pressure, pre-eclampsia…	Covered in indication 1 of “abnormal progression of labor”.
2. Fetal causes: - Macrosomia	Macrosomia often leads to “prolonged labor” or “shoulder dystocia”, which are covered in the new indications.
- Abnormal presentations: occipital posterior position, face presentation, breech presentation	Occipital posterior position may be associated with caesarean, vacuum, or forceps, which are covered in indication 3. Face presentation often leads to prolonged labor, which is covered in indication 1. Breech presentation often leads to prolonged labor or fetal distress, which are covered in indication 1.
- Premature, fetal distress	Episiotomy is only needed in the case of fetal distress, covered in indication 2.
3. Instrumental delivery (forceps, vacuum, breech presentation)	Covered in indication 3 of “vacuum or forceps childbirth”.

## Data Availability

Data presented in this project report was provided by Phu Vang District Health Center; Quang Nam Province General Hospital; Hung Vuong Hospital; and Quang Ninh Obstetrics and Pediatrics Hospital. Requests for data may be directed to the corresponding author and are subject to institutional data use agreements.

## Supplementary Materials

The following supporting information can be downloaded at: https://www.mdpi.com/article/10.3390/healthcare11182520/s1. Table S1: Monthly Episiotomy Data Report Form; S2: Letter to MOH on the revision of episiotomy policy.

## Appendix A

**Table A1:** List of participating hospitals.

No.	Province	Hospital	Average Annual Births	Type of Hospital				
				National or Provincial	District	ObGyn/ObPed	Private	Data Included in This Report
1	Ca Mau	Ca Mau Maternity—Pediatric Hospital	8300	X		X		
2		Tran Van Thoi Area General Hospital	2000		X			
3	Hue	Phu Vang District Health Center	1300		X			X
4	Quang Nam	Vinh Duc General Hospital	12,700		X		X	
5		Minh Thien General Hospital	9500		X		X	
6		Quang Nam Province General Hospital	5000	X				X
7	Da Nang	Da Nang Hospital for Women and Children	14,300	X		X		
8	Can Tho	Can Tho Obstetrics and Gynecology Hospital	15,000	X		X		
9		Phuong Chau International General Hospital	5000	X			X	
10	Ho Chi Minh	Tu Du Hospital	65,000	X		X		
11		Hung Vuong Hospital	42,000	X		X		X
12	Quang Ninh	Quang Ninh Obstetrics and Pediatrics Hospital	6800	X		X		X
Total			186,900	8	4	6	3	4
