# Supplementary material for: Promoting Respectful Maternity Care by Reducing Unnecessary Episiotomies: Experiences from Centers of Excellence for Breastfeeding in Vietnam
Source: Healthcare (Basel). 2023 Sep 12;11(18):2520. doi: 10.3390/healthcare11182520 (PMC10530948; doi:10.3390/healthcare11182520)
Supplement: Supplementary file 1 [file healthcare-11-02520-s001.zip › Supplementary Material S2- Letter to MOH on the revision of episiotomy policy_ENG.pdf]

Hanoi, November 25, 2019

To: Dr Nguyen Duc Vinh, Director General Department of Maternal and Child Health, Ministry of Health

Cc: Tu Du Hospital, Hung Vuong Hospital, Danang Hospital of Obstetric and Pediatric, Quang Ninh Obstetrics and Pediatrics Hospital, Vinh Duc Hospital, Minh Thien Hospital, Quang Nam Provincial General Hospital, Phu Vang District Hospital, Can Tho Obstetrics and Gynecology Hospital, Phuong Chau Hospital, Ca Mau Obstetrics and Pediatrics Hospital, Tran Van Thoi Hospital

Re: Selective episiotomy policies

Alive & Thrive would like to send our sincerest appreciation to the Ministry of Health, Departments of Health and hospitals for promoting breastfeeding in Viet Nam. By reviewing charts during Center of Excellence for Breastfeeding assessments at 26 hospitals in April and November 2019, we found that 92% of cases (n=280) conducted episiotomy, which is not recommended by the World Health Organization for routine or liberal use.

From 7 to 22 November 2019, Alive & Thrive collaborated with Ina Landau Aasen from Oslo University Hospital (Norway) to conduct four courses on Friendly Delivery Room and Skilled Birth Attendance with 120 TOT trainers from 12 hospitals of 7 provinces nationwide. The trainer conducted clinical coaching on the hands-on technique with ten vaginal deliveries at four hospitals. All participating hospitals committed to adopting selective episiotomy, monitoring and sharing monthly data with Alive & Thrive from December 2019.

With this correspondence, Alive & Thrive would like to inform the Department of Maternal and Child Health and recommend updating the National Guideline on Episiotomy hereupon.

On February 2018, WHO issued recommendation on episiotomy and perineal trauma prevention as follows:

> ***RECOMMENDATION 38.*** *For women in the second stage of labor, techniques to reduce perineal trauma and facilitate spontaneous birth (including perineal massage, warm compresses and a "hands on" guarding of the perineum) are recommended, based on a woman's preferences and available options.*[1]
>
> ***RECOMMENDATION 39.*** *Routine or liberal use of episiotomy is not recommended for women undergoing spontaneous vaginal birth*[2].

Evidence summary: A Cochrane systematic review that included 12 RCTs on 6177 women in Argentina, England, Canada, Germany, Spain, Ireland, Malaysia, Pakistan, Columbia and Saudi

---

[1] WHO Reproductive Health Library. WHO recommendation on techniques for preventing perineal trauma in second stage of labor (February 2018). The WHO Reproductive Health Library; Geneva: World Health Organization.
[2] WHO Reproductive Health Library. WHO recommendation on episiotomy policy (February 2018). The WHO Reproductive Health Library; Geneva: World Health Organization

Arabia shows that for women where unassisted vaginal birth was anticipated, routine episiotomy is not justified to reduce perineal trauma. Routine episiotomy is associated with an increase of third- and fourth-degree tears and subsequent anal sphincter muscle dysfunction[3]. Selective episiotomy policies result in fewer women with severe perineal/vaginal trauma[4].

Selective Episiotomy: According to WHO's clinical practice pocket guide on early essential newborn care, which was translated into Vietnamese and endorsed by the Department of Maternal and Child Health (December 2014)[5], the episiotomy guideline is as follows:

- *Do not perform routine episiotomy.*
- *Episiotomy should be considered only in the case of:*
    - *complicated vaginal delivery (breech, shoulder dystocia, vacuum or forceps extraction);*
    - *scarring of the female genitalia or poorly healed third- or fourth-degree tears; or*
    - *fetal distress.*
- *Provide good perineal support with controlled delivery of the head.*

According to WHO's "Introducing and sustaining EENC in hospitals: routine childbirth and newborn care" (2016)[6], four restricted episiotomy indications which must be written in medical record include:

1. *Abnormal progression of labor*
2. *Non-reassuring fetal heart rate pattern*
3. *Vacuum or forceps delivery*
4. *Shoulder dystocia*

Regarding the definition "Abnormal progression of labor", please refer to WHO's recommendation on physiology of labor (2018)[7] as follows:

The first stage of labor:

> ***RECOMMENDATION 8.*** *A minimum cervical dilatation rate of 1 cm/hour throughout active first stage is unrealistically fast for some women and is therefore not recommended for identification of normal labor progression. A slower than 1-cm/hour cervical dilatation rate alone should not be a routine indication for obstetric intervention. (Not recommended).*
>
> ***RECOMMENDATION 9.*** *Labor may not naturally accelerate until a cervical dilatation threshold of 5 cm is reached, therefore the use of medical interventions to accelerate labor and birth (such as oxytocin augmentation or caesarean section) before this threshold is not recommended, provided fetal and maternal conditions are reassuring. (Not recommended)*

The second stage of labor:

---

[3] WHO, Managing complications in pregnancy and childbirth: a guide for midwives and doctors – 2nd ed. 2017
[4] https://www.ncbi.nlm.nih.gov/pmc/articles/PMC5449575/
[5] https://web.wpro.who.int/firstembrace/wp-content/uploads/2017/08/img-downloads-country_publications-eenc_clinical_pocket_guide_vnm.pdf
[6] https://iris.wpro.who.int/bitstream/handle/10665.1/13409/9789290617808-eng.pdf?ua=1
[7] WHO recommendations: Intrapartum care for a positive childbirth experience, 2018, p. 51

RECOMMENDATION 33. *The second stage is the period of time between full cervical dilatation and birth of the baby, during which the woman has an involuntary urge to bear down, as a result of expulsive uterine contractions. (Recommended)*

Women should be informed that the duration of the second stage varies from one woman to another. In first labors, birth is usually completed within 3 hours whereas in subsequent labors, birth is usually completed within 2 hours. (Recommended)

RECOMMENDATION 19. *For woman with epidural analgesia in the second stage of labor, delaying pushing for one to two hours after full dilatation or until the woman regains the sensory urge to bear down.*

We enclose herewith the detailed recommendation on episiotomy indications in the annex. We hope that it serves as a reference for the Ministry of Health to revise the Decision 1377/QD-BYT dated April 24, 2013 on National technical guideline for examination and treatment in Gynecology and Obstetrics. For more information, please contact Ms. Vu Hoang Duong, Technical Specialist, Alive & Thrive Southeast Asia, 091.456.8011, vduong@fhi360.org.

Yours Sincerely,

Roger Mathisen

Regional Director

Alive & Thrive Southeast Asia

APPENDIX. RECOMMENDATION ON SELECTIVE EPISIOTOMY POLICIES

Evidence summary

A Cochrane systematic review that included 12 RCTs on 6177 women in Argentina, England, Canada, Germany, Spain, Ireland, Malaysia, Pakistan, Columbia and Saudi Arabia shows that for women where unassisted vaginal birth was anticipated, routine episiotomy is not justified to reduce perineal trauma. Routine episiotomy is associated with an increase of third- and fourth-degree tears and subsequent anal sphincter muscle dysfunction[i]. Selective episiotomy policies result in fewer women with severe perineal/vaginal trauma [ii].

In 2018, WHO issued recommendation on episiotomy and perineal trauma prevention on global scale as follows:

> ***RECOMMENDATION 38.*** *For women in the second stage of labor, techniques to reduce perineal trauma and facilitate spontaneous birth (including perineal massage, warm compresses and a "hands on" guarding of the perineum) are recommended, based on a woman's preferences and available options*[iii].
>
> ***RECOMMENDATION 39.*** *Routine or liberal use of episiotomy is not recommended for women undergoing spontaneous vaginal birth*[iv].

Guideline on episiotomy

1. Episiotomy:
   a) Avoid routine episiotomy
   b) Selective episiotomy indications:
      1. *Abnormal progression of labor*
      2. *Non-reassuring fetal heart rate pattern*
      3. *Vacuum or forceps delivery*
      4. *Shoulder dystocia*
   c) Episiotomy indications must be written in the medical record
2. Definition of abnormal progression of labor:
   a) Primiparous women with or without epidural anesthesia: > 3 hours
   b) Multiparous women with or without epidural anesthesia: > 2 hours

Hospitals evaluate their situation to apply this definition.

Perineal support techniques during childbirth – "hands-on" technique

1. One hand slowering the delivery of the head
2. Other hand protecting perineum
3. Mother not pushing when head is crowning
4. Episiotomy by indication only

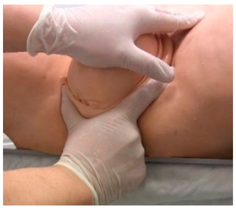

Table. Monthly Episiotomy Data

Month	Primiparous women							Multiparous women						
	CM (N)	CM (%)	RM (N)	RM (%)	NV (N)	NV (%)	Total (N, 100%)	CM (N)	CM (%)	RM (N)	RM (%)	NV (N)	NV (%)	Total (N, 100%)
1														
2														
3														

CM (Cắt may) = Episiotomy; RM (Rách may) = Natural tear; NV (Nguyên vẹn) = No tear

COMPARISION BETWEEN THE SELECTIVE EPISIOTOMY INDICATIONS AND DECISION 1377/QD-BYT

(dated April 24, 2013 on National technical guideline for examination and treatment in Gynecology and Obstetrics)

Through the below analysis, we would like to emphasize that the above proposed recommendation has fully and more clearly reflected the indication in the decision 1377/QD-BYT. Details as below:

DECISION 1377/QD-BYT	ALIVE & THRIVE'S RECOMMENDATION
**1. Mother causes:**<br>- due to prolonged labor or many vaginal examinations	"The perineum is thick, hard, swelling" is a vague indication. In many cases, it is due to the limited waiting time. A&T proposes to delete the "thick, hard, swelling perineum" indication and keeps the indication of "prolonged labor", which has been defined in the indication of "abnormal progression of labor".
- Mothers' diseases: heart failure, high blood pressure, pre-eclampsia…	Covered in the indication of "abnormal progression of labor".
**2. Fetus causes:**<br>- Macrosomia	Macrosomia often leads to "prolonged labor" or "shoulder dystocia", which are covered in the new indication lists.
- Abnormal presentations: occipital posterior position, face presentation, breech presentation	Occipital posterior position may be associated with caesarean, vacuum or forceps[v], which are covered in the new indication lists.<br>Face presentation often leads to prolonged labor, which is covered in the new indication lists.<br>Breech presentation often leads to prolonged labor, fetal distress, which are covered in the new indication lists.
- Premature, fetal distress	Episiotomy is only needed for premature with fetal distress, which have been covered in the new indication lists.
**3. Instrumental delivery (forceps, vacuum, breech presentation)**	Covered in the indication of "vacuum, forceps delivery".

[i] https://www.ncbi.nlm.nih.gov/pmc/articles/PMC5449575/

[ii] WHO, Managing complications in pregnancy and childbirth: a guide for midwives and doctors – 2nd ed. 2017

[iii] WHO, Managing complications in pregnancy and childbirth: a guide for midwives and doctors – 2nd ed. 2017

[iv] WHO Reproductive Health Library. WHO recommendation on episiotomy policy (February 2018). The WHO Reproductive Health Library; Geneva: World Health Organization

[v] WHO, Managing complications in pregnancy and childbirth: a guide for midwives and doctors – 2nd ed. 2017
