# Supplementary material for: Promoting Respectful Maternity Care by Reducing Unnecessary Episiotomies: Experiences from Centers of Excellence for Breastfeeding in Vietnam
Source: Healthcare (Basel). 2023 Sep 12;11(18):2520. doi: 10.3390/healthcare11182520 (PMC10530948; doi:10.3390/healthcare11182520)
Supplement: Supplementary file 1 [file healthcare-11-02520-s001.zip › Supplementary Table S1-Monthly Episiotomy Data Report Form.pdf]

Monthly Episiotomy Data Report Form

**Month ……**	**Nulliparous women**							**Multiparous women**						
**Day**	Episiotomy		Natural tear		No tear (N)		Total	Episiotomy		Natural tear		No tear		Total
	n	%	n	%	n	%	n	n	%	n	%	n	%	n
**1**														
**2**														
**3**														
**4**														
**5**														
**6**														
**7**														
**8**														
**9**														
**10**														
**…**														
**30**														
**31**														
**Total**														
